# Rapid visuomotor feedback gains are tuned to the task dynamics

**DOI:** 10.1152/jn.00748.2016

**Published:** 2017-08-23

**Authors:** Sae Franklin, Daniel M. Wolpert, David W. Franklin

**Affiliations:** ^1^Computational and Biological Learning Laboratory, Department of Engineering, University of Cambridge, Cambridge, United Kingdom;; ^2^Institute for Cognitive Systems, Department of Electrical and Computer Engineering, Technical University of Munich, Munich, Germany; and; ^3^Neuromuscular Diagnostics, Department of Sport and Health Sciences, Technical University of Munich, Munich, Germany

**Keywords:** adaptation, learning, motor control, reaching movement, visuomotor feedback

## Abstract

Adaptation to novel dynamics requires learning a motor memory, or a new pattern of predictive feedforward motor commands. Recently, we demonstrated the upregulation of rapid visuomotor feedback gains early in curl force field learning, which decrease once a predictive motor memory is learned. However, even after learning is complete, these feedback gains are higher than those observed in the null field trials. Interestingly, these upregulated feedback gains in the curl field were not observed in a constant force field. Therefore, we suggest that adaptation also involves selectively tuning the feedback sensitivity of the sensorimotor control system to the environment. Here, we test this hypothesis by measuring the rapid visuomotor feedback gains after subjects adapt to a variety of novel dynamics generated by a robotic manipulandum in three experiments. To probe the feedback gains, we measured the magnitude of the motor response to rapid shifts in the visual location of the hand during reaching. While the feedback gain magnitude remained similar over a larger than a fourfold increase in constant background load, the feedback gains scaled with increasing lateral resistance and increasing instability. The third experiment demonstrated that the feedback gains could also be independently tuned to perturbations to the left and right, depending on the lateral resistance, demonstrating the fractionation of feedback gains to environmental dynamics. Our results show that the sensorimotor control system regulates the gain of the feedback system as part of the adaptation process to novel dynamics, appropriately tuning them to the environment.

**NEW & NOTEWORTHY** Here, we test whether rapid visuomotor feedback responses are selectively tuned to the task dynamics. The responses do not exhibit gain scaling, but they do vary with the level and stability of task dynamics. Moreover, these feedback gains are independently tuned to perturbations to the left and right, depending on these dynamics. Our results demonstrate that the sensorimotor control system regulates the feedback gain as part of the adaptation process, tuning them appropriately to the environment.

## Abstract (precis)

Here, we test whether rapid visuomotor feedback responses are selectively tuned to the task dynamics. The responses do not exhibit gain scaling, but they do vary with the level and stability of task dynamics. Moreover, these feedback gains are independently tuned to perturbations to the left and right, depending on these dynamics. Our results demonstrate that the sensorimotor control system regulates the feedback gain as part of the adaptation process, tuning them appropriately to the environment.

we constantly interact with our environment, be it by playing sports or drinking a cup of coffee. To produce skilled movements, unaffected by these dynamic interactions, we need to predict the task dynamics and adapt our control strategy accordingly. It has been shown that the sensorimotor control system builds a predictive feedforward controller of the internal and external dynamics (Conditt et al. 1997; Goodbody and Wolpert 1998; Kluzik et al. 2008; Lackner and Dizio 1994; Shadmehr and Mussa-Ivaldi 1994). This feedforward controller predictively generates the appropriate pattern of muscle activation that compensates for the dynamics of the environment and generalizes these predictions across a variety of kinematics and limb states. A sudden change in task dynamics during the movement causes kinematic errors, leading to large increases in muscle cocontraction (Franklin et al. 2003; Thoroughman and Shadmehr 1999) and feedback gains (Cluff and Scott 2013; Franklin et al. 2012). These reactive responses act to limit the perturbing effects of the new dynamics until the sensorimotor control system is able to learn a new internal model, or adapt the previous model, that can predictively compensate for this change in dynamics. Once the internal model is updated, the reactive responses are gradually decreased (Franklin et al. 2012) along with the cocontraction (Franklin et al. 2003).

Feedback corrections to errors during reaching can arise through both muscle stretch-dependent motor responses (stretch reflexes) (Bennett 1994; Kurtzer et al. 2009; Nashed et al. 2014) and rapid visuomotor responses responding to shifts in the visual location of the hand (Franklin and Wolpert 2008; Sarlegna et al. 2003; Saunders and Knill 2003), the target (Brenner and Smeets 1997; Day and Lyon 2000; Goodale et al. 1986; Hayashi et al. 2016; Oostwoud Wijdenes et al. 2011), or the visual background (Abekawa and Gomi 2015; Saijo et al. 2005). These visuomotor responses are involuntary in nature (Day and Lyon 2000; Franklin and Wolpert 2008; Gomi 2008), with loop delays to force production on the order of 150 ms (Franklin et al. 2016). Insights into the control of such involuntary responses may provide important insight into voluntary control (Franklin and Wolpert 2011), as it has been suggested that the same neural circuitry underlying such rapid motor responses is also involved in voluntary control (Pruszynski et al. 2011a), as proposed by the optimal feedback control framework (Scott 2004; Todorov 2004; Todorov and Jordan 2002).

In our previous work, we proposed that the large changes in rapid visuomotor feedback gains during initial learning resulted from the increased uncertainty in the internal model (Franklin et al. 2012). We demonstrated increased rapid visuomotor feedback gains early in curl force field learning, which decreased once the predictive motor memory was learned. However, even after learning, these feedback gains remained high compared with those in null field trials. Moreover these upregulated feedback gains in the curl field were not observed with constant background loads. We propose that these final levels of feedback gains were not simply increased according to the uncertainty, but were actually adapted and tuned to the task dynamics. We propose that the changes in feedback gain seen in this previous work highlight two computational components of feedback modulation: reactive control and predictive control. The reactive control produces an initial (likely generalized) increase in feedback gains in response to uncertainty in the environment (Franklin et al. 2012) and parallels the rise in cocontraction (Franklin et al. 2003, 2012; Darainy and Ostry 2008; Osu et al. 2002). In contrast, the predictive controller gradually tunes and adapts the feedback gains appropriately for the environmental dynamics as learning proceeds (Cluff and Scott 2013). Therefore, adaptation does not only involve learning a set of predictive muscle activation patterns but also learning to selectively tune the feedback sensitivity of the sensorimotor control system to the environment. This is supported by studies showing that stretch-dependent feedback responses are modified for movements in stable (Ahmadi-Pajouh et al. 2012; Cluff and Scott 2013; Wagner and Smith 2008) and unstable dynamics (Franklin et al. 2007), although some results are confounded by gain scaling (Pruszynski et al. 2009). Similarly visuomotor feedback responses have been show to modify with changes in the visuomotor mapping (Franklin and Wolpert 2008; Franklin et al. 2014; Hayashi et al. 2016). However, these studies have examined feedback modulation under a limited set of experimental conditions, such as a single force field, making it difficult to determine the degree to which the feedback gains can be modulated. Here, we expand upon these results, examining how the visuomotor feedback gains adapt to different characteristics of the environmental dynamics. Specifically we examine how the predictive visuomotor feedback gains scale across a broader range of background loads, different types of force fields, and, in particular, for force fields with asymmetric dynamics. That is, we examine whether or not these learned feedback responses can match the asymmetry of the environmental dynamics.

## MATERIALS AND METHODS

Seventeen subjects provided written informed consent to participate in the experiments, which were approved by the Cambridge Psychology Research Ethics Committee. All subjects were right-handed, according to the Edinburgh handedness inventory (Oldfield 1971), with no reported neurological disorders. Subjects were allocated to the three experiments (*n* = 8, 10, and 8), in which each subject participated in either one or two of the experiments.

### Apparatus

**Fig. 1.**
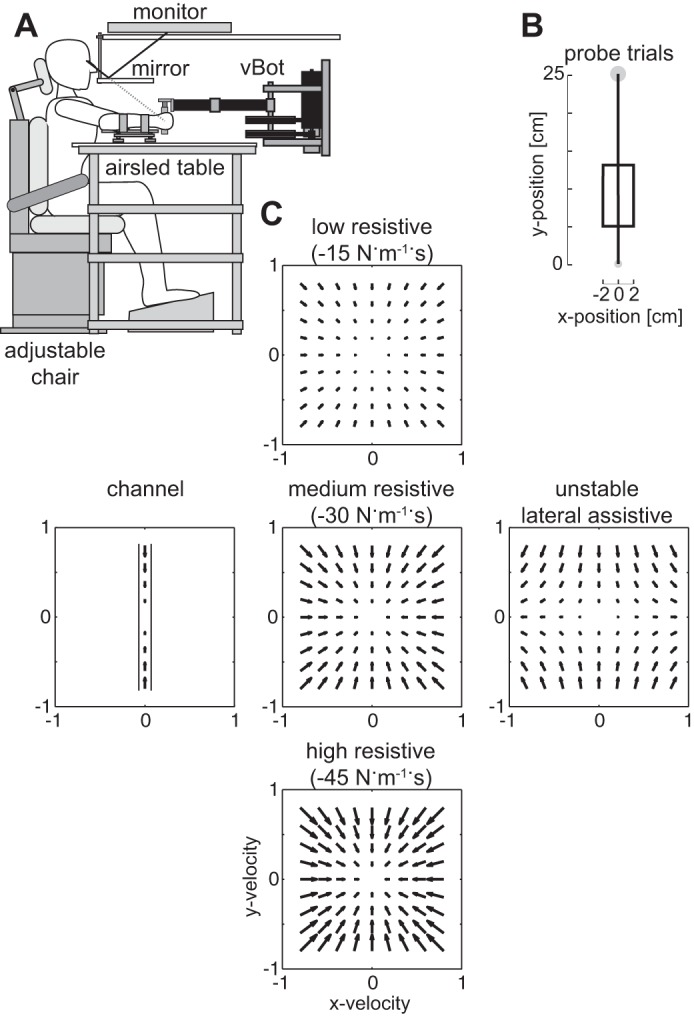
Experimental setup. *A*: seated subject grasped the robotic manipulandum (vBOT), while visual feedback was presented veridically using a top-mounted monitor viewed through a mirror. The subject’s forearm was supported by an airsled. *B*: visual perturbations (probe trials) used to examine the magnitude of the rapid visuomotor feedback responses in *experiments 1* and *2*. On randomly selected reaching trials, the hand was constrained physically to a straight line to the target (mechanical channel), while the visual representation of the hand (cursor) was jumped laterally away from the actual hand location for 250 ms before being returned. *C*: five force fields used in *experiment 2* are shown as vector fields as a function of hand velocity. The central column shows the three resistive force fields that resist motion in any direction in proportion to velocity (*top*: low resistance; *middle*: medium resistance; *bottom*: high resistance). The central row shows the three force fields with identical resistance in the direction of movement, but which vary in the resistance in the direction orthogonal to movement (*left*: mechanical channel; *middle*: medium resistance; *right*: unstable assistance).

Subjects were seated with their shoulders restrained against the back of a chair by a shoulder harness and grasped the handle of the vBOT robotic manipulandum (Howard et al. 2009) with their forearm supported against gravity with an air sled (Fig. 1*A*). The robotic manipulandum both generated the environmental dynamics (null field, force field, or channel) and measured the subjects’ behavior. Position and force data were sampled at 1 kHz. End point forces at the handle were measured using an ATI Nano 25 six-axis force-torque transducer (ATI Industrial Automation, Apex, NC). The position of the vBOT handle was calculated from joint-position sensors (58SA; IED) on the motor axes. Visual feedback was provided using a computer monitor mounted above the vBOT and projected veridically to the subject via a mirror. This virtual reality system covers the manipulandum, arm and hand of the subject, preventing any visual information about their location. The exact time that the stimuli were presented visually to the subjects was determined using the video card refresh rate and confirmed with an optical sensor to measure any time delays. Subjects performed right-handed forward reaching movements in the horizontal plane at ~10 cm below their shoulder level.

### Experimental Setup

Movements were made from a 1-cm diameter start circle centered ~28 cm in front of the subject to a 2-cm diameter target circle centered 25 cm in front of the start circle. The subject’s arm was hidden from view by the virtual reality system, which displayed the start and target circles, as well as a 0.6-cm diameter cursor representing the hand position. A successful movement required the hand cursor to enter the target (without overshooting) within 700 ± 75 ms of movement initiation. Overshoot was defined as movements that exceeded the target in the direction of movement. When subjects performed a successful movement, they were provided with feedback of how close they were to the desired movement time of 700 ms (“great” if within ± 37.5 ms, otherwise “good”) and the counter increased. When they performed unsuccessful movements, they were provided with feedback as to why the movement was not considered successful (“too fast”, “too slow,” or “overshot target”), and the counter remained at the previous value. All trials were recorded regardless of their success. The initiation of trials was self-paced; subjects initiated a trial by moving the hand cursor into the start circle and holding it within the target for 1,000 ms. A tone then indicated that the subjects could begin the movement to the target. The duration of the movement was determined from the time that the hand cursor exited the start circle until the time that the cursor entered the final target.

### Probe Trials To Measure Feedback Gains

To assess rapid visuomotor feedback magnitude, visually induced motor responses were examined using perturbations of the visual system similar to those previously described (Dimitriou et al. 2013; Franklin and Wolpert 2008; Franklin et al. 2016; Reichenbach et al. 2014). On random probe trials, when the hand had moved a specific percentage of the distance to the target (e.g., 20% or 5 cm), the cursor representing the hand position was jumped away from the current hand position, held 2 cm away laterally from the actual hand trajectory for 250 ms and then returned to the actual hand position for the rest of the movement (Fig. 1*B*). The direction of the jump (left vs. right) was randomized across trials. During these trials, the hand was physically constrained to the straight path between the initial starting position and the final target using a mechanical channel, such that any force produced in response to the visual perturbation can be measured against the channel wall using the force sensor. The mechanical wall of the channel was implemented with a stiffness of 5,000 N/m and damping of 10 N·m^−1^·s for any movement lateral to the straight line joining the starting location and the middle of the target (Milner and Franklin 2005; Scheidt et al. 2000). As this visual perturbation is transitory with the cursor returning to match the actual hand position, subjects are not required to respond to this visual perturbation to produce a successful trial. These visual perturbations were applied perpendicular to the direction of the movement (either to the left or the right). For comparison, a zero perturbation trial was also included, in which the hand was held to a straight-line trajectory to the target but the visual cursor remained at the hand position throughout the trial. The perturbation trials were randomly applied during movements in a blocked fashion, such that one of each of the three perturbations was applied within a block of trials. A nonprobe trial movement was always performed first in any new phase, such that a probe trial was never the first movement.

### Experimental Paradigm

Three experiments were performed to examine whether the rapid visuomotor feedback gains are adapted to the dynamics of the external environment after adaptation.

#### Experiment 1: visuomotor gains under a resistive background load.

Eight subjects (two female, six male: aged 29.1 ± 6.7, means ± SD) participated in this experiment, in which we extend our previous finding (Franklin et al. 2012) that the rapid visuomotor feedback gain does not increase with an externally applied constant background load. Previously, we applied forces orthogonal to the line between the start and target locations. In this experiment, the background load was applied in the direction opposite to the movement, which is along the line joining start and target location. The three visual perturbation or probe trials (rightward +2.0 cm, zero, or leftward −2.0 cm) were presented pseudo-randomly within a single block of nine trials (three probe trials and six visually unperturbed trials) to assess the visuomotor response. The onset of the displacements occurred at 5 cm from the start of the movement (20% of the length of the movement). Each probe trial was repeated 30 times for each background force level.

In the experiment, on every trial, a constant force was applied in the direction opposite (−*y*) to the direction of movement. The constant force was experienced at six levels (3, 5, 7, 9, 11, and 13 N), where all of the trials at a particular force level were blocked together. The order of the blocks of constant forces was randomized across subjects. Subjects performed the experiment in two sessions in which three of the force levels were experienced in each session. For each force level, 271 trials (of which 90 were probe trials) were performed. Subjects were required to take short breaks every 100 movements throughout the experiment. They were also allowed to rest at any point they wished. To initiate a trial, subjects moved into the start circle, and then, the background load ramped up over 300 ms. Once the desired background load was achieved and subjects had stabilized their hand within the start circle for 1,000 ms, a tone indicated that the subject should perform the reaching movement to the target. Once subjects had maintained the hand within the target circle for 400 ms, the background force was ramped back down over 300 ms. Throughout the movement, the background force level and direction were constant in Cartesian space.

#### Experiment 2: visuomotor gains under a viscous force field.

Ten subjects participated in the second experiment (three female, seven male: aged 28.5 ± 6.1) examining the role of viscous force fields on the rapid visuomotor feedback gains. Our previous work (Franklin et al. 2012) demonstrated that the introduction of a viscous force field had two effects: an initial increase in feedback gains related to the magnitude of the kinematic error and an increased final level of feedback gain relative to the level in the null field. This second increase in the feedback gain after extensive learning was proposed to arise through an adaptation of the feedback gain to the increased uncertainty in the environment due to the interaction between signal-dependent noise and a velocity-dependent force field (Franklin et al. 2012). This possibility arises as muscle activation increases during adaptation causing an appropriate increase in motor noise. Both motor noise and planning noise (Churchland et al. 2006; van Beers 2009) would cause an increase in the trial-by-trial variability. The actual forces produced by the force field depend entirely on the specific trajectory performed; thus, variability in the trajectory increases the variability in the applied forces, increasing the uncertainty of the environment and state of the limb. To examine whether the final plateau level of feedback gains is truly adapted to the environmental dynamics, we examined the final adaptation to five different environmental dynamics. Subjects adapted to each force field in a randomized order across subjects in a single session on one day. The background force field was modulated in two ways relative to a baseline-resistive force field: the magnitude of the field and degree of stability. The baseline force field was a resistive viscous field:

$$\left[\begin{array}{c} F_{x}\\ F_{y} \end{array}\right]=\left[\begin{array}{cc} -b & 0\\ 0 & -b \end{array}\right]=\left[\begin{array}{c} \overset{˙}{x}\\ \overset{˙}{y} \end{array}\right]$$

where *b* was 30 N·m^−1^·s (Fig. 1*C*, *middle*).

To examine the degree to which the rapid visuomotor feedback response scaled with the magnitude of the force field, the constant *b* was changed for two environments: either decreased to 15 N·m^−1^·s or increased to 45 N·m^−1^·s (Fig. 1*C*, *top* and *bottom*). All three force fields are stable; however, each force field would be expected to influence the visuomotor reflex differently if the sensorimotor control system adapts to the visuomotor reflex to the task dynamics. Specifically, the resistance in the direction orthogonal to the movement increases; therefore, if the hand had been actually perturbed by the size of the visual perturbation, then a smaller or larger amount of restoring force is required to bring the hand back to the original movement for the lower and higher resistive force fields, respectively.

To examine the effect of stability in the external environments, two further force fields were examined where the stability was only manipulated in the direction orthogonal to the forward reaching movement. The stable force field had the addition of a mechanical channel in the lateral direction with stiffness of 5,000 N·m^−1^ and viscosity of 10 N·m^−1^·s, with no change in the forward reaching direction (Fig. 1*C*, *left*). Specifically, this was implemented as

$$\left[\begin{array}{c} F_{x}\\ F_{y} \end{array}\right]=\left[\begin{array}{cc} -b & 0\\ 0 & -10 \end{array}\right]\left[\begin{array}{c} \overset{˙}{x}\\ \overset{˙}{y} \end{array}\right]+\left[\begin{array}{cc} -5,000 & 0\\ 0 & 0 \end{array}\right]\left[\begin{array}{c} x\\ y \end{array}\right]$$

where the value of *b* was the same as for the baseline field (30 N·m^−1^·s). The unstable field had an assistive viscous element in the direction orthogonal to the direction of motion (Fig. 1*C* right) and was implemented as

$$\left[\begin{array}{c} F_{x}\\ F_{y} \end{array}\right]=\left[\begin{array}{cc} -b & 0\\ 0 & +10 \end{array}\right]\left[\begin{array}{c} \overset{˙}{x}\\ \overset{˙}{y} \end{array}\right]$$

where the value of *b* was the same as for the baseline field (30 N·m^−1^·s).

As in the first experiment, subjects performed 271 trials in each condition, comprising 90 probe trials (30 rightward, 30 zero, and 30 leftward probes) and 181 trials in the specific force field. In all probe trials, while the lateral forces were constrained by the channel, the forces in the direction of the movement were those of the condition (e.g., resistive viscous field). Although lateral movement in the random probe trials was constrained by the mechanical channel, the subjects were free to move in any manner during all of the other trials. All other conditions were the same as in the first experiment, except that subjects did not need to wait for a background force to be ramped up or down at the beginning or end of each trial.

#### Experiment 3: rapid visuomotor feedback gains for asymmetric fields.

Eight subjects participated in the third experiment (six female, two male: aged 23.5 ± 3.8) investigating whether the visuomotor feedback gain could be independently modulated for leftward and rightward perturbations if the force field produced different forces to leftward and rightward motion. Our previous work (Franklin et al. 2014) has shown that the late visuomotor feedback gains can be independently modulated to leftward and rightward perturbations when different task-relevant or task-irrelevant sensory discrepancies are applied to the left or right of the movement. Here, we further investigate this issue to determine whether these visuomotor feedback gains also modulate independently to leftward or rightward perturbations, according to the dynamics of the environment. Three different environmental conditions were studied, which varied in terms of the lateral viscous component, all of which were implemented as

$$\begin{array}{l} \left[\begin{array}{c} F_{x}\\ F_{y} \end{array}\right]=\left[\begin{array}{cc} {\begin{array}{c} b \end{array}}_{1} & 0\\ 0 & 0 \end{array}\right]\left[\begin{array}{c} \overset{˙}{x}\\ \overset{˙}{y} \end{array}\right]\text{if}\;\overset{˙}{x}\geq 0\\ \left[\begin{array}{c} F_{x}\\ F_{y} \end{array}\right]=\left[\begin{array}{cc} b_{2} & 0\\ 0 & 0 \end{array}\right]\left[\begin{array}{c} \overset{˙}{x}\\ \overset{˙}{y} \end{array}\right]\text{if}\;\overset{˙}{x}<0 \end{array}$$

**Fig. 2.**
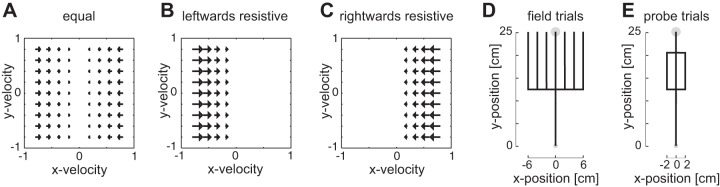
Experimental setup for *experiment 3*. *A*: equal resistive force field is matched in resistance (proportional to velocity) to motions to the right or left of the reaching movement but does not resist motion in the direction of the movement. *B*: leftward resistive field resists motion only in the leftward direction proportional to the velocity. *C*: rightward resistive field resists motion only in the rightward direction. *D*: on normal reaching movements in each of the three fields, the visual location of the hand was shifted to one of seven locations between −6 and 6 cm at the halfway point of the movement. Subjects had to correct for this shift and bring the cursor into the target by the end of the movement. *E*: visual perturbations (probe trials) used to examine the magnitude of the rapid visuomotor feedback responses. On random reaching trials, the hand was constrained physically to reach in a straight line toward the target (mechanical channel), while the visual representation of the hand (cursor) was jumped laterally away from the actual hand location for 250 ms before being returned. The onset of this perturbation was the halfway point of the movement.

The equal condition was implemented as *b_1_* = *b_2_* = −20 (N·m^−1^·s), such that the field provided equal resistance to movements with positive or negative *x-*velocity and no resistance in the *y-*axis (Fig. 2*A*). The strong leftward resistance condition (Fig. 2*B*) was implemented as *b_1_* = 0 and *b_2_* = −40 (N·m^−1^·s), whereas the strong rightward resistance condition (Fig. 2*C*) was implemented as *b_1_* = −40 and *b_2_* = 0 (N·m^−1^·s). Subjects were split randomly into two equally sized groups, where both groups started with the equal resistance condition. One group then performed the strong rightward resistance condition followed by the strong leftward condition, whereas the other group performed these two in the opposite order.

Each condition consisted of 50 blocks, where each block consisted of 10 trials (500 trials total). As in the previous two experiments, each block consisted of three probe trials (rightward +2 cm, zero or leftward −2 cm) presented pseudo-randomly in which lateral motion was constrained by a mechanical channel (Fig. 2*E*). The other trials in the block consisted of trials in which the visual cursor was shifted laterally by one of seven magnitudes [−6.0, −4.0, −2.0, 0.0, 2.0, 4.0, and 6.0] cm and held at this distance for the remainder of the trial (Fig. 2*D*). This ensured that subjects experienced the forces that are not apparent if they made a perfectly straight movement to the target. Therefore, the subjects were required to compensate for the imposed visual displacement to bring the cursor into the target by the end of the movement (Franklin et al. 2016). All probe trials and maintained visual perturbations occurred at 12.5 cm from the start position (50% of the movement distance). The onset of the visual perturbations was set to the middle of the movement, as the rapid visuomotor feedback gain is highest at this point during the movement (Dimitriou et al. 2013), as predicted by optimal feedback control theory (Liu and Todorov 2007). The cursor was 1.0 cm in diameter, start circle was 1.4 cm in diameter, and target circle was 1.6 cm in diameter. Short rest breaks were required every 200 trials, although movements were self-paced throughout the experiment, allowing subjects to take breaks at any point. All other details were matched to *experiment 2*.

### Analysis

Analysis of the experimental data was performed using MATLAB R2015a. Position, velocity, and end point force were low-pass filtered at 40 Hz with a fifth-order, zero-phase lag Butterworth filter. Acceleration was calculated by differentiating the filtered velocity. Repeated-measures ANOVAs were performed in MATLAB using the ranova function. If a significant main effect of force field was found, planned multiple comparisons between the responses in each of the force fields (multcompare function) were performed using the Tukey-Kramer method. When appropriate, linear regression was performed for each subject using the mean responses for each condition. The slopes across subjects were then examined using a Student's *t*-test to determine whether the slopes were significantly different from zero. Statistical significance was considered at the *P* < 0.05 level for all statistical tests.

The purpose of the study was to examine the relation between the task dynamics and the feedback gains after adaptation. To examine adaptation to the task, we calculated several kinematic and descriptive parameters over the training period. For each measure, we calculated the mean across all force fields or loads as a function of the block number. The results were used to determine the period over which these measures became stable, so that we could use this period to examine the feedback gains after learning. On the basis of this analysis, we omitted the first five blocks, as these measures changed rapidly during this phase. After removing the first five blocks of trials, the mean response (and standard error of the mean) for each condition was calculated and plotted.

#### Hand path error.

The maximum perpendicular error (MPE) of the hand was used as a measure of the straightness of the hand trajectory. The MPE is the maximum distance on the actual trajectory that the hand reaches perpendicular to the straight-line path joining the start and end circles (errors to the left are defined as negative, and errors to the right are defined as positive). The MPE was calculated for each nonprobe trial throughout the learning experiment.

#### Success rate.

Each movement was designated a successful trial if the subject performed the movement within the desired time (700 ± 75 ms) and did not overshoot the target.

#### Movement duration.

The movement duration was calculated as the time between the subject leaving the start circle and first entering the target circle as long as they maintained their position within the final target circle for 400 ms. If the subject passed through the target, overshooting the target completely, then the duration included this overshoot up to the point at which subjects entered the target and were able to stabilize within the target.

#### Peak velocity.

The peak velocity was calculated as the maximum velocity in the direction of movement (*y*-axis) that occurred between the subject leaving the start circle and first entering the target circle.

#### Rapid visuomotor responses.

Individual probe trials were aligned on visual perturbation onset. The response to the right visual perturbation (or left visual perturbation) on probe trials was subtracted from the response on zero probe trials to provide two estimates of the motor response to the visual perturbation for each block. Depending on the experiment, these were either averaged (*experiments 1* and *2*) or analyzed separately (*experiments 1* and *3*). Visuomotor responses from the first five blocks of each experiment were not used in the analysis. To examine the feedback gain, we calculated the average postperturbation force over two intervals: the first corresponding to a rapid involuntary response (180–230 ms) (Franklin and Wolpert 2008), and the second to a slower response (230–300 ms), which may be a mixture of involuntary and voluntary responses. The early interval was conservatively determined (Franklin and Wolpert 2008) using a voluntary reaction task (Day and Lyon 2000) to determine an interval that avoided any voluntary responses.

For the third experiment, receiver operator characteristic (ROC) analysis (Pruszynski et al. 2008) was performed to determine the earliest time at which visuomotor responses were modulated independently for perturbations in the force fields. Specifically, to examine whether there was independent modulation of the feedback responses for different force fields and to determine the time that such independent modulation occurs, we generated an ROC curve for every 1-ms sample. That is, we calculated the area under the ROC (aROC) curves for the ability to distinguish between the responses to the same perturbation in the rightward resistive field and the leftward resistive field. The discrimination time was taken as the point when the aROC exceeds 0.75 for three consecutive samples. As we are interested in the time point at which this difference emerges in the force responses, we examine the time point where the information begins to deviate from chance (Thompson et al. 1996). To do this, we excluded aROC after the discrimination point and fit a dog leg to the aROC data (flat line at aROC of 0.5 followed by a linear component). The time of the end of the flat portion of the fit was taken as the onset time of the response (Pruszynski et al. 2008). The ROC analysis was performed using the individual data for each subject separately, as well as across the subjects using the mean traces for each subject.

## RESULTS

We examined the modulation of visuomotor responses to environmental dynamics in three separate experiments. In each experiment, the background dynamics were modulated to test whether the rapid visuomotor feedback gains modulate across these changes in the environment. In each dynamical environment, subjects performed reaching movements while grasping the handle of a robotic manipulandum (Fig. 1*A*). The rapid visuomotor feedback gains were then measured on randomly selected trials (termed probe trials) during which the visual cursor, representing the hand position, was perturbed while the physical hand was mechanically constrained to move within a channel to the target. These visuomotor perturbations were orthogonal to the channel either to the left or right (Figs. 1*B* and 2*E*) and resulted in an involuntary motor response producing force against the channel wall. This change in lateral force was quantified over appropriate temporal windows to estimate the feedback gain.

### Experiment 1. Visuomotor Gains Under a Background Load

**Fig. 3.**
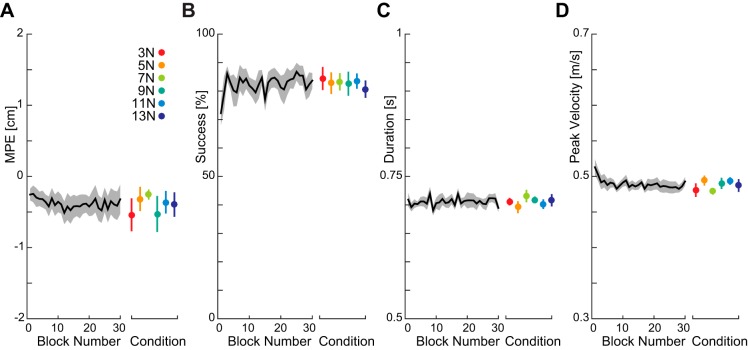
Measures of adaptation to the six background loads (*experiment 1*). The background force was changed every 30 blocks (271 trials), and the order of the forces was randomized across subjects. The black lines indicate the mean measure, collapsed across different background loads to show learning within a background load block. *A*: maximum perpendicular error of the hand (MPE) collapsed across the six background loads (means ± SE across subjects) as a function of block number. The colored error bar plot shows the MPE (means ± SE across subjects for last 25 blocks) for each background load separately. *B*: success rate as a function of block number. *C*: movement duration across blocks. The desired duration was 0.700 ± 0.075 s. *D*: peak velocity.

The first experiment was designed to further examine changes in rapid visuomotor feedback gains that might occur with a constant background load. In a previous study, we showed that constant lateral forces produce no increase in the rapid visuomotor feedback gain over a small range of force levels (Franklin et al. 2012). Here, we used probe trials to measure the rapid visuomotor feedback responses with different levels of constant background force (3, 5, 7, 9, 11, and 13 N), where the forces were applied along the direction of motion that is opposite to motion and, therefore, orthogonal to the forces in our previous study. This direction of background load corresponds to the direction of increased loading due to the resistive fields used in *experiment 2*. Subjects rapidly learned to produce consistent movements with the background load with minimal kinematic errors and a high success rate (Fig. 3). Figure 3 shows that subjects performed stably after the first 5 blocks when a new background force was introduced (Fig. 3, black lines). Therefore, we used the last 25 blocks to examine the visuomotor gain, which showed little variance between conditions (Fig. 3, colored error bars).

**Fig. 4.**
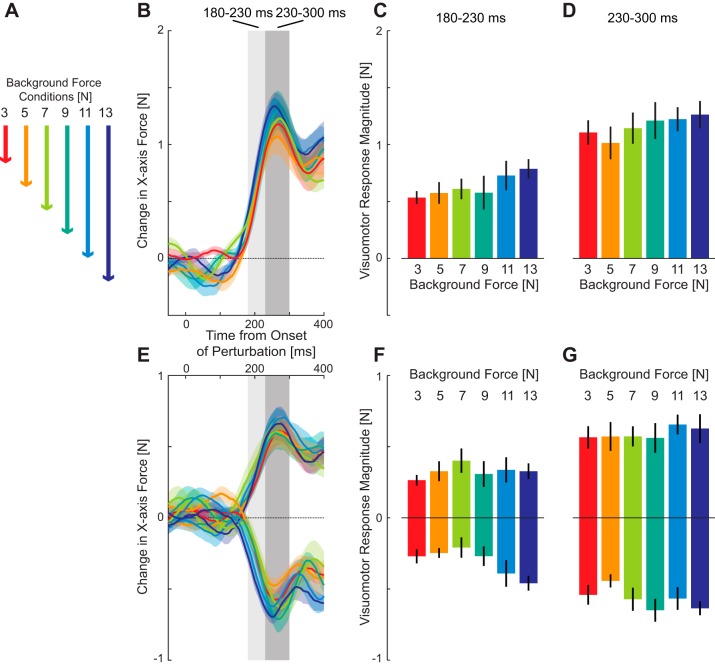
Rapid visuomotor feedback responses during reaching with a constant background load (*experiment 1*). *A*: in each condition, one of six different levels of resistive force was applied to the subject’s hand by the robotic system throughout the movement. *B*: mean force responses to visual perturbations (difference in force produced on left and right perturbation on probe trials) for each of the six force levels (means ± SE across subjects) are shown aligned to the onset of the perturbation. The color corresponds to the background load. The gray regions illustrate the time windows over which the force response was quantified. *C*: means ± SD force response over the early time window (180–230 ms) corresponding to an involuntary period. *D*: means ± SD force response over the late time window (230–300 ms). *E*: mean force responses to visual perturbations (probe trials) for left (positive traces) and right (negative traces) perturbation directions plotted separately. *F*: means ± SD force response over the early window by perturbation direction for left and right perturbations. *G*: means ± SD force response over the late window by perturbation direction.

The response to the perturbation shows clear force responses for all six force levels (Fig. 4*A*) with a peak in the force around 250 ms after the onset of the perturbation (Fig. 4*B*). Although there were no dramatic differences in the force traces, the highest background force levels appeared to have the slightly larger force responses. This was investigated by determining the mean visuomotor force response over both the initial involuntary feedback window (180–230 ms) and a later interval (230–300 ms) (Fig. 4, *B* and *C*). The results of a repeated-measures ANOVA with main factor of condition (six levels) showed no significant main effect of force level for either the early (*F*_5,35_ = 1.44; *P* = 0.233) or late (*F*_5,35_ = 0.971; *P* = 0.448) intervals.

Similar to previous studies (Saijo et al. 2005), we also performed linear regression on the mean data for each subject separately to examine gain scaling. We compared the slopes across subjects with a *t*-test to determine whether the slopes were significantly different from zero. The slopes for the early interval (0.0241 ± 0.031; means ± SD) were not significantly different than zero (*t*_7_ = 2.21; *P* = 0.063). This was also true to the late interval, where the slopes (0.0211 ± 0.036; mean ± SD) were not significantly different than zero (*t*_7_ = 1.62; *P* = 0.15). Therefore, despite more than a fourfold increase in background force level, there were no significant differences in the rapid visuomotor feedback gain across the conditions. This remained true, even when the perturbations to the left and the perturbations to the right were separately examined (Fig. 3, *E*–*G*) within a single repeated-measures ANOVA with main factors of condition (six levels) and perturbation direction (two levels). We found no significant main effect of condition (*F*_5,35_ = 1.444; *P* = 0.233), perturbation direction (*F*_1,7_ = 0.560; *P* = 0.479), or interaction effect (*F*_5,35_ = 1.430; *P* = 0.238) for the early interval. Similarly, we found no significant main effect of condition (*F*_5,35_ = 0.971; *P* = 0.449), perturbation direction (*F*_1,7_ = 0.163; *P* = 0.699), or interaction effect (*F*_5,35_ = 1.071; *P* = 0.393) for the late interval. Overall, there were no significant differences in the magnitude of the response to leftward and rightward perturbations, regardless of loading conditions.

These results, combined with our previous finding (Franklin et al. 2012), suggests that the rapid visuomotor feedback responses do not exhibit the gain scaling to background force, unlike the short latency stretch reflex responses (Pruszynski et al. 2009). In particular, the resistive force fields in the next experiment (*experiment 2*) require larger forces in the direction of motion. Here, we show that higher forces in this direction produce limited effects on the visuomotor feedback responses and, therefore, that any differences are unlikely to be explained by this factor.

### Experiment 2. Visuomotor Gains Under a Viscous Force Field

**Fig. 5.**
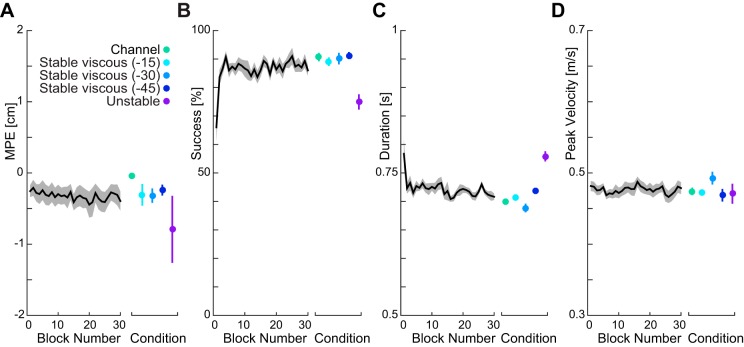
Measures of adaptation to the five viscous force fields (*experiment 2*)—same format as Fig. 3. The black lines indicate the mean measure, collapsed across different conditions, to show the change within the time of the experiment. Error bars are means ± SE across the last 25 blocks in each force field. *A*: maximum perpendicular error (MPE) (means ± SE) across the five fields. *B*: success rate. *C*: movement duration. *D*: peak velocity.

Our previous study found that even after learning a velocity-dependent force field, the rapid visuomotor feedback gain was increased relative to that seen in a null force field (Franklin et al. 2012), which suggested that the rapid visuomotor feedback gains might be adapted to the environmental dynamics. The second experiment tested this possibility by investigating whether the rapid visuomotor feedback responses scaled with changes in the magnitude and type of force field. Subjects adapted to three different levels of a resistive viscous force field (Fig. 1*C*, *middle column*; −15, −30 and −45 N·m^−1^·s), as well as two force fields where the stability was manipulated in the direction orthogonal to the reach direction (Fig. 5*C*, left and right fields). Stable performance in terms of kinematic error and duration were found by the fifth block of trials (Fig. 5), so we again analyzed the last 25 blocks. As expected, there were differences between the conditions on these measures (Fig. 5, colored error bars), with the unstable condition having the lowest success rate and largest MPE. However the peak velocity was similar across all conditions.

**Fig. 6.**
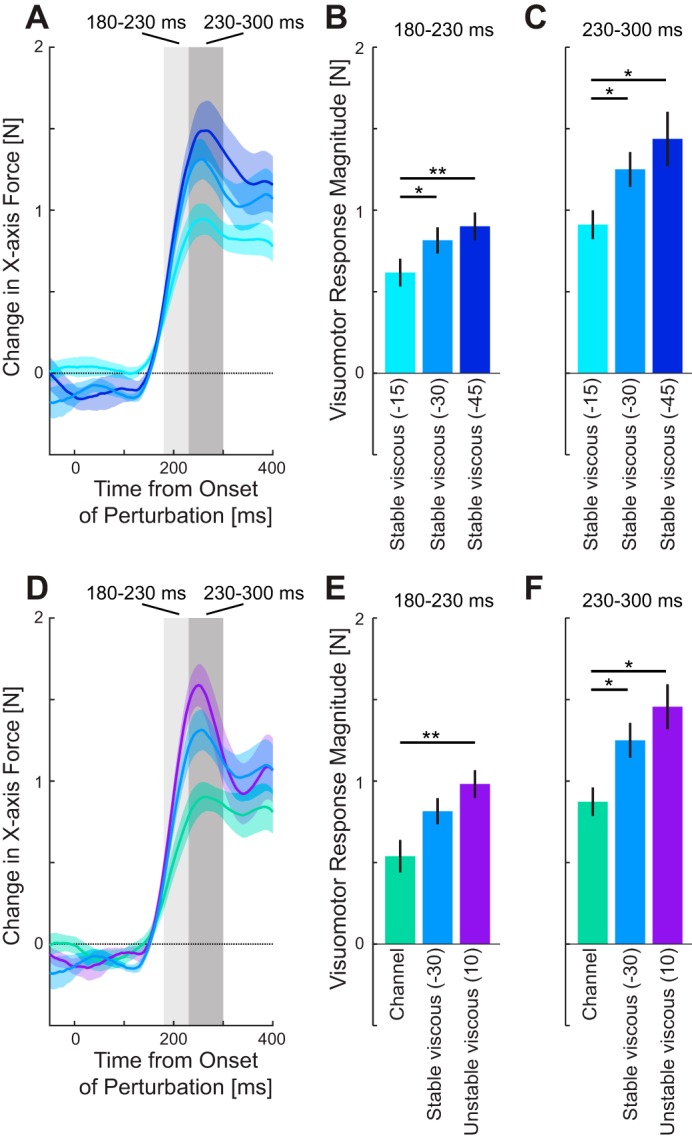
Rapid visuomotor feedback responses during reaching in viscous force fields (*experiment 2*). *A*: mean force response (± SE) on probe trials for the three levels of resistive viscous force fields (−15: light blue; −30: blue; −45: dark blue). *B*: means ± SD force response over the early time window (180–230 ms) corresponding to an involuntary period for the fields in A. *C*: means ± SD force response over the late time window (230–300 ms). *D*: force response on probe trials in the three force fields with different lateral stability conditions (mechanical channel: green; resistive viscous field −30 N·m^−1^·s: blue; assistive viscous field +10 N·m^−1^·s: purple). *E*: mean ± SD force during the early time window. *F*: means ± SD force response during the late time window. Statistically significant differences between the conditions were tested with the Tukey-Kramer multiple-comparisons test (*P < 0.05; **P < 0.01).

As the resistive viscosity increased, the feedback response on the probe trials also increased (Fig. 6*A*, light to dark blue). The magnitude of these responses was examined over the two intervals using an ANOVA with main effect of force field and random effect of subjects. There was a significant increase in the feedback response for both the early (*F*_2,18_ = 6.799; *P* = 0.006) and late (*F*_2,18_ = 9.273; *P* = 0.002) intervals. After significant main effects, the post hoc comparisons indicated that the feedback responses were significantly different for the −15 N·m^−1^·s field compared with both the −30 (*P* = 0.046) and −45 N·m^−1^·s (*P* = 0.009) fields, but there was no significant difference between the two highest force fields (*P* = 0.64) during the early interval (Fig. 6*B*). This effect was maintained in the late interval, with significant differences between the −15 N·m^−1^·s field and the −30 (*P* = 0.011) and −45 N·m^−1^·s (*P* = 0.011) fields, and no difference (*P* = 0.394) between the two highest fields (Fig. 6*C*). However, similar to *experiment 1*, we performed linear regression for each subject. In contrast to the previous experiment, here, we found that the slopes between the force response and the force field value were significantly different from zero for both the early (*t*_9_ = 3.88; *P* = 0.0037) and late intervals (*t*_9_ = 3.77; *P* = 0.0044). Thus as the resistive force field increased in strength, the visuomotor response gain also increased.

Three conditions (Fig. 1*C*, *middle row*) in the experiment were matched in terms of the forces in the forward direction with a resistive viscous force of −30 N·m^−1^·s (the middle field above). One field (Fig. 1*C*, *middle row*) was uniformly resistive in all directions, while the other two varied in the stability in the direction orthogonal to movement. The more stable fields were constrained to always be in a mechanical channel, while the less stable field was assistive in the orthogonal direction and, hence, unstable (+10 N·m^−1^·s). Despite the same forward resistance to motion, the feedback responses showed strong differences in their amplitudes (Fig. 6*D*). Again, there were significant main effects of force field on the feedback gains for both the early (*F*_2,18_ = 8.869; *P* = 0.002) and late (*F*_2,18_ = 9.841; *P* = 0.0013) time intervals. Post hoc comparisons indicated that the feedback response in the mechanical channel was significantly smaller than the unstable force field (*P* = 0.003), but there were no differences between the resistive fields responses and the unstable (*P* = 0.11) or channel fields (*P* = 0.12) for the early interval (Fig. 6*E*). At the later window, the channel was significantly different from both the resistive (*P* = 0.028) and unstable (*P* = 0.015) force fields (Fig. 6*F*).

### Experiment 3. Rapid Visuomotor Feedback Gains for Asymmetric Fields

**Fig. 7.**
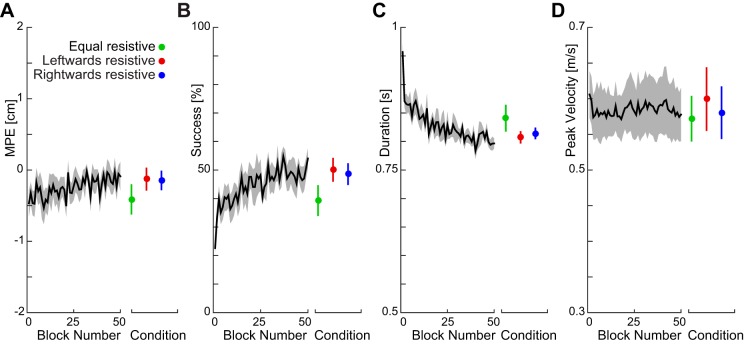
Measures of adaptation to the equal, leftward resistive, and rightward resistive fields (*experiment 3*). The black lines indicate the mean measure, collapsed across different conditions, to show the change within the time of the experiment. Error bars are means ± SE across the last 45 blocks in each force field. *A*: MPE (means ± SE). *B*: success rate. *C*: movement duration. *D*: peak velocity.

The previous experiment showed that the rapid visuomotor feedback responses vary appropriately depending on the force field in which subjects made their movements. As the first experiment demonstrated that constant forces and, therefore, simple changes in background load, do not affect the feedback gains, the results of the second experiment suggested that the rapid visuomotor feedback gains adapt to the force field to provide an appropriate compensation for the dynamics. In the third experiment, we test this possibility directly by introducing force fields that only have a lateral component orthogonal to the direction of movement. Specifically, we examine the feedback gains in three force fields in which the appropriate feedback response to a perturbation to the left or right of straight reaching movement would vary. The fields were either equal on both sides of the reach direction, strongly resistive to leftward motion, or strongly resistive to rightward motion (Fig. 2, *A*–*C*). Subjects made reaching movements in all three fields in a blocked design. In each field, along with probe trials to measure feedback gains, on nonprobe trials, the cursor was shifted laterally to one of seven locations (ranging from −6 to +6 cm), and the subjects were required to compensate for the shift by the end of the movement, so that the cursor entered the target (Fig. 2*D*). These visual shifts were included to ensure participants experienced the lateral forces over the workspace. It is important to note that the majority of the trials are ones in which a cursor jump is present. The velocity-dependent resistive force fields, therefore, make it more difficult to return the cursor to the midline. While the maximum perpendicular error and peak velocity changed little throughout the adaptation, the success rate increased and the duration decreased (Fig. 7). It can be seen from the figure that the major effect on performance was within the first five blocks, with a much more gradual improvement in performance after these initial movements. The first five blocks of trials were, therefore, not analyzed in terms of the visuomotor gain. Differences between the conditions were small (Fig. 7, colored error bars).

**Fig. 8.**
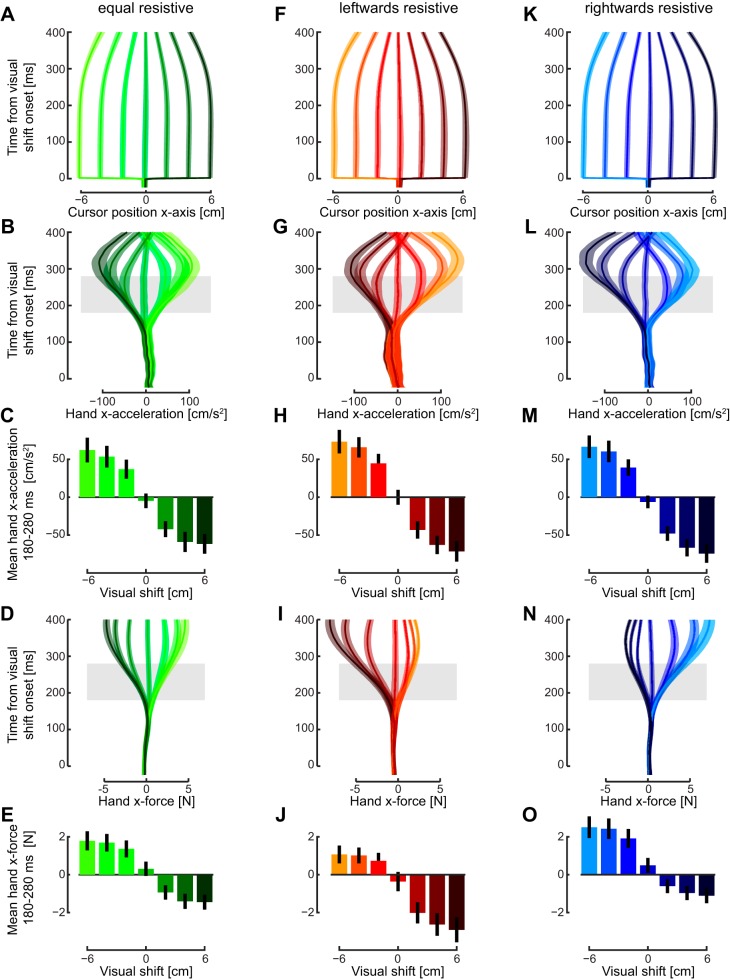
Corrective responses on the nonprobe trial movements in the equal (*left column*), leftward resistive (*middle column*) and rightward resistive (*right column*) fields (*experiment 3*). *A*: lateral (*x*-axis) cursor position as a function of the time from the visual shift that occurred at half of the movement distance. The means ± SE across subjects is shown for each of the seven visual shifts ranging from −6 cm (leftward shift: light green) to +6 cm (rightward shift: dark green). *B*: lateral acceleration of the hand (means ± SE) for each of the seven visual shifts as a function of time from shift onset. *C*: means ± SD of the lateral hand acceleration between 180 and 280 ms after the onset of the visual shift. *D*: lateral force produced by the hand (means ± SE) for the seven visual shifts. *E*: means ± SD of lateral hand force between 180 and 280 ms after visual shift onset. *F–J*: same as *A–E* for the leftward resistive force field (rightward shifts: dark red; leftward shifts: light red). *K–O*: same as *A–E* during the rightward resistive force field (rightward shifts: dark blue; leftward shifts: light blue).

The majority of trials (70%) were ones in which the cursor was shifted laterally by up to 6 cm, and subjects had to compensate for the shift, bringing the cursor toward the target. However, in each field, the required force to produce this action varied, particularly for movements rightward or leftward. Adaptation, therefore, required subjects to produce different amounts of corrective force in the three force fields. We start by examining the corrective responses of these trials to shifts in the cursor. After a cursor jump, subject made corrective movement under all fields, bringing the cursor back toward the target (Fig. 8, *A*, *F*, and *K*). In the symmetric force field, the lateral acceleration in response to the shift in visual hand position increased proportional to the magnitude and direction of the shift (Fig. 8*B*). The onset of the change in acceleration started ~150 ms after the visual shift, a response time equivalent to the normal visuomotor feedback delays (Franklin and Wolpert 2008; Franklin et al. 2016; Reichenbach et al. 2009). The mean acceleration was then examined over a 100-ms interval (180–280 ms). It can be seen that the kinematic responses to leftward and rightward shifts were approximately equal as expected (Fig. 8*C*). These roughly equivalent responses in terms of kinematics were also seen for the leftward-resistive (Fig. 8, *G* and *H*) and rightward-resistive force fields (Fig. 8, *L* and *M*). However, if the kinematics were similar but force fields are different, then the sensorimotor system must have changed the force response to a given size of visual shift. This was investigated by looking at the lateral hand force (measured with the force transducer at the handle) in each of the three fields (Fig. 8, *D*, *I*, and *N*). It is apparent that the early force responses are adapted appropriately to the force fields with increased force responses to rightward perturbations in the leftward resistive field (Fig. 8*J*) with no accompanying increase in the response to leftward perturbations (larger magnitude dark reds compared with light reds). Note that a rightward cursor perturbation requires a leftward compensatory motion to get to the target, and, therefore, the subject would experience the high resistive force in this direction for this force field. The opposite response (large forces in response to leftward perturbations) is seen in the rightward resistive force field (Fig. 8*O*, larger responses for light blue compared with dark blue). Finally, in the equal resistive field, the responses to perturbations in either direction were similar (Fig. 8*E*). These results show that the corrective responses have been appropriately adapted to each of the three force fields over the initial 100 ms of response. In particular, it also demonstrates that these corrective responses can be independently controlled to the right or left of the straight reaching movement.

**Fig. 9.**
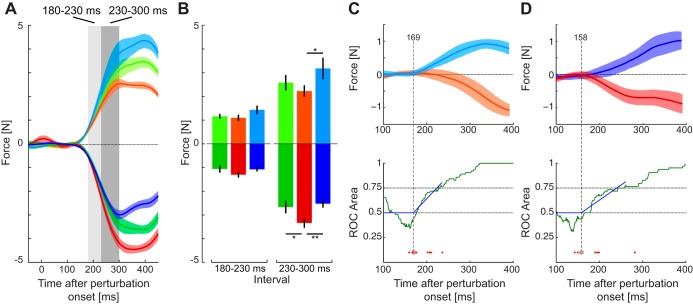
Rapid visuomotor feedback responses during reaching in the equal, leftward resistive, and rightward resistive fields (*experiment 3*). *A*: mean force response (± SE) on probe trials in the three force fields (equal: green; leftward resistive: red; rightward resistive: blue) for the rightward (dark colors) and leftward (light colors) visual perturbations. Responses have been subtracted from the zero perturbation condition in each force field. *B*: means ± SD of the force response over the early and late time windows. *C: top*: difference in force responses to the leftward perturbations for the rightward (light blue) and leftward resistive force fields (orange) relative to the equal force field. The difference between these two measures was examined using receiver operator characteristic (ROC) analysis to determine the time point at which the signals could be discriminated by an ideal observer. *Bottom*: vertical axis represents the probability that an ideal observer could discriminate between the responses in the two force fields. The green curve is the area under the ROC based on each subject’s mean response. The solid blue line illustrates the dog leg fit which is used to determine the onset of the significant difference (large red circle). ROC analysis was also performed separately for each subject (small red circles). *D*: differences in force responses for the rightward perturbations and the associated ROC analysis.

However, the previous results are based on measurements of the kinematics and force responses in freely moving trials, making it difficult to examine the exact time course of the corrective responses. Therefore, throughout the experiments, subjects were also presented with probe trials (brief visual shifts with a mechanical channel resisting changes in lateral motion) to measure the feedback gains. The force responses to these probe perturbations were examined relative to a zero perturbation condition for all three force fields (Fig. 9*A*). Over the full time period, clear differences could be seen in the responses, with movements in the block with the leftward resistive field (red), showing larger responses to rightward perturbations than the equal (green) or rightward (blue) resistive fields. For leftward perturbations the responses were reversed with the leftward resistive field showing the small responses (orange), followed by the equal condition (light green) and largest response in the rightward resistive field (light blue). The mean feedback force was then quantified over both the early and late intervals (Fig. 9*B*). In the early interval, while it appeared there may be an effect of the force field, the main effect of force field in the ANOVA failed to reach significance for either perturbations to the right (*F*_2,14_ = 2.966; *P* = 0.084) or left (*F*_2,14_ = 2.915; *P* = 0.087) of the reaching movement. However, for the later interval (Fig. 9*B*) there were significant main effects for perturbations to the right (*F*_2,14_ = 11.159; *P* = 0.0013) and to the left (*F*_2,14_ = 8.431; *P* = 0.004). Post hoc tests found significant differences between the rightward and leftward fields for both the rightward perturbation (*P* = 0.007) and the leftward perturbation (*P* = 0.025). For both the right and left perturbations, we found significant slopes (*t*_7_ = 4.498; *P* = 0.003 and *t*_7_ = 3.461; *P* = 0.011, respectively) at the late interval, but only for the right perturbation at the early interval (*t*_7_ = 2.962; *P* = 0.021).

These analyses showed that the modulation of the responses to the perturbations are changed by the late interval; however, it did not allow us to examine at what time point they become significantly different. We used ROC analysis (Pruszynski et al. 2008) to look at the time point when the responses each perturbation direction in the rightward and leftward resistive fields could be distinguished relative to the baseline equal condition (see methods). For the leftward perturbation (Fig. 9*C*), this occurred at 169 ms (large red circle) and for rightward perturbations (Fig. 9*D*), this occurred at 158 ms (large red circle). We also calculated the onset times for each subject individually (small circles). The estimated time point at which the significant difference begins were consistently earlier than the earliest measure of voluntary feedback (230 ms) that was seen for a single subject in a previous study (Franklin and Wolpert 2008) and far before the estimated voluntary response time (265 ms) for similar perturbations (Kobak and Mehring 2012). This suggests that complex patterns of feedback gains, which vary on one-side of a reaching movement compared with the other, can be controlled according to the dynamics of the environment within involuntary time windows.

## DISCUSSION

The adaptation of rapid visuomotor feedback gains to temporal changes in the environmental dynamics was examined during reaching movements. We found that the magnitude of these feedback gains was not affected by increases in a constant background load (over a fourfold range) opposing the direction of movement. However, in a second experiment, the feedback gains scaled strongly with changes in the viscous environment, increasing as the resistive force field increased. Moreover, the feedback gains also varied as the lateral component alone changed, increasing in a laterally unstable field, while decreasing when stability and lateral accuracy were guaranteed (mechanical channel). These results suggested that the rapid visuomotor feedback gains adapt to the environment as part of the learning process. To examine this, we conducted a third experiment in which we examined this effect more precisely by introducing force fields that vary in the required responses to perturbation to the right or left of a reaching movement. The results showed that the relative feedback responses to leftward and rightward perturbations are clearly changed in the late interval period, with initial differing responses occurring within the involuntary time window. These modulated feedback responses were appropriately modified to the dynamics of the external environment, and larger when higher resistive forces would have been present. Overall, our results demonstrate that dynamic adaptation not only involves learning the predictive feedforward control of muscle activity but also involves the tuning of feedback gains to the novel environment.

While the short latency stretch reflex responses have long been known to exhibit scaling with background muscle activity or automatic gain scaling (Bedingham and Tatton 1984; Matthews 1986; Pruszynski et al. 2009), this was not found for rapid visuomotor feedback responses elicited by shifts in the visual hand position (Franklin et al. 2012). Here, we further examined this issue by using larger background loads (up to 13 N constant load) in the direction of reaching. Again, we found little change in the feedback magnitude over the wide range of constant loads examined, with responses of a similar magnitude to those of our previous study. This further suggests that these responses do not exhibit such gain scaling. In short latency stretch reflexes, the gain scaling is thought to result from the organization of the motoneuron pool where the motor units are recruited, according to their force-generating capability or size (Capaday and Stein 1987; Henneman 1957; Marsden et al. 1976). However, later responses gradually show reduced gain scaling (Pruszynski et al. 2009) until there is no effect for steady-state voluntary control (Milner-Brown and Stein 1975), suggesting that the sensorimotor control system compensates for the nonlinear recruitment of the motoneuron pool. This decrease in the gain scaling is well matched with the timing of increased cortical contributions to the long latency responses (Pruszynski et al. 2011a, 2011b) and the subsequent sophistication of these feedback responses (Kurtzer et al. 2008; Nashed et al. 2014). The absence of gain scaling in the visuomotor responses also suggests that a similar model of the motoneuron pool recruitment process must be used to adapt the commands to the underlying background muscle activity. However, it is not clear whether this arises through cortical processing of the motor commands, as some evidence has suggested a subcortical pathway for the earliest visuomotor responses through the colliculus (Reynolds and Day 2012). It is important to note that in our experiments, we have only examined the force responses (feedback gains) as a function of the background load, rather than examine the muscular responses using electromyography. While this technique may miss subtle effects that could be seen in the muscle activity, it also provides a comprehensive overall response of all muscles that could be responding to the perturbation. In contrast, analysis of the electromyographic activity only samples a subset of the muscles and motor units that contribute to the overall response. Although we cannot claim a definitive test of the gain-scaling within this paper, it is clear that larger forces in the direction of motion have limited effects on the visuomotor feedback gain. In particular, a fourfold change in load produced at most a 20% (not significant) increase in the rapid visuomotor response. Thus we can claim that any changes seen in *experiment 2* are unlikely to be affected by the small changes in loading that occur within these studies.

The absence of any evidence of gain scaling for visuomotor responses contrasts with the results for the manual following response, or MFR (Abekawa and Gomi 2015; Gomi et al. 2006; Saijo et al. 2005). It has been shown that the MFR exhibits stronger responses when the limb is loaded (Saijo et al. 2005), similar to the gain scaling of stretch reflex responses. In particular, they demonstrated significant correlations between the MFR and the background load and muscle activity for a variety of loading conditions. In contrast, our results showed no significant change in gain with respect to background load. Although we only examined this with eight subjects, similar numbers of subjects demonstrated highly significant slopes in all other experiments. This raises an intriguing question as to whether these two visuomotor feedback responses arise through distinct pathways, which could explain these differences in the scaling of the response.

In this study, we examined whether the sensorimotor control system is able to tune the rapid visuomotor feedback responses to environmental dynamics as part of the adaptation process, that is, to tune them to the environment to provide part of the adaptation to the dynamics. Several previous studies have shown evidence that feedback responses are modulated depending on the environment (Ahmadi-Pajouh et al. 2012; Cluff and Scott 2013; Diamond et al. 2015; Franklin et al. 2007, 2012; Kimura and Gomi 2009; Kobak and Mehring 2012; Krutky et al. 2010; Wagner and Smith 2008; Yousif and Diedrichsen 2012). Some of these studies have shown that feedback responses to physical perturbations after adaptation elicit responses that appear to be suitable for the change in environmental dynamics, but the measurements could not be separated from voluntary responses (Franklin et al. 2007; Wagner and Smith 2008) or limb admittance (Yousif and Diedrichsen 2012). Moreover, many of these studies involve changes in the background muscle activity, which means that any change in feedback gain is difficult to dissociate from the effect produced by gain scaling. To avoid these issues, a recent study measured the feedback responses by applying perturbations before the start of movement to examine the feedback component alone (Ahmadi-Pajouh et al. 2012), finding changes in the long latency feedback gain in the preparatory period before the movement, similar to the finding that gains are affected by the decision process (Selen et al. 2012). However, changes in the feedback responses during the postural phase before movement initiation does not indicate that the feedback gains are utilized as part of the adaptation to the dynamic environment. To address all of these issues, Cluff and Scott (2013) utilized a novel approach to the experimental design. They had subjects adapt to a force field in two directions, such that no adaptation was necessary in a third movement located in the middle. Despite no changes in the background activity for movements in this middle movement direction, the long latency feedback gains were increased as subjects adapted to the force fields, with the feedback gain peaking at the end of the adaptation process. Another approach, which we use here, is to study rapid visuomotor feedback responses during adaptation (Franklin et al. 2012; Kobak and Mehring 2012), as these do not exhibit gain scaling.

These previous studies have shown that feedback gains are changed after force field adaptation or learning of novel dynamics. However, only a few of these studies have been able to clearly demonstrate that the change in the feedback responses are appropriate for the change in the environmental dynamics, with differential feedback gains for different environments. Cluff and Scott (2013) showed that the size of the stretch reflex was modified with the size of the viscous load learned in other parts of the workspace. Later, it was shown (Diamond et al. 2015) that subjects adjusted their grip force according to the learned dynamics of the environment when visuomotor perturbations of the hand location or target were imposed. Here, we expand on these results by modifying the resistive force of the background force field. In *experiment 2*, we introduced three levels of resistive viscosity, which meant that lateral perturbations would require larger or smaller force responses to return the hand to the unperturbed trajectory. The feedback responses were appropriately increased or decreased according to the dynamics, suggesting that adaptation also involved the tuning of the feedback gains to the dynamics. We recently showed that rapid visuomotor feedback gains can be independently modulated to leftward and rightward perturbations when different task-relevant or task-irrelevant sensory discrepancies are applied to the left or right of the movement (Franklin et al. 2014). More recently, it has been shown that visuomotor reflexes elicited through target jumps are also modulated by learning a distorted sensorimotor map imposed through visuomotor rotations (Hayashi et al. 2016). We extended this work by demonstrating that feedback adaptation to environmental dynamics also exhibited such independent modulation, where the feedback responses were tuned differentially to rightward and leftward perturbations. Moreover, the relative increase and decrease in feedback responses were appropriate for the changes in the force field, which only acted laterally to the direction of the movement. Therefore, our work extends previous results by clearly demonstrating that the visuomotor feedback responses are tuned within the involuntary window to the dynamics of the environment. What is not yet clear, however, is whether this adaptation of the feedback responses is part of the adapted feedforward motor memory (Wagner and Smith 2008) or is an independent and dissociable mechanism of adaptation (Yousif and Diedrichsen 2012).

We suggest that there are at least two computational components to the measured feedback gains during adaptation to novel dynamics. Visuomotor feedback responses during adaptation to a curl force field showed an initial rapid increase in feedback gains that was gradually reduced to a lower plateau as the subjects adapted to the force field (Franklin et al. 2012). Thus, after the initial increase, the feedback gains were reduced as the predictive model was learned. However, in a later study it was shown that stretch reflexes are gradually modified during learning, increasing in parallel to the predictive model (Cluff and Scott 2013). While these two results may initially appear conflicting, we propose that they highlight two computational components of feedback modulation: reactive control and predictive control. Faced with uncertainty about the environment, the sensorimotor control system upregulates a (likely default) pattern of feedback gains—what we term the reactive feedback system. This rise in feedback gains parallels the increase in cocontraction (Franklin et al. 2003, 2012). However, even in these first movements, the sensorimotor system is already learning the dynamics, gradually tuning the predictive controllers (Milner and Franklin 2005; Sing et al. 2009), including predictive feedback gains to the environment. Here, we have shown further evidence that these predictive feedback gains can be tuned appropriately to the dynamics, even differentially tuned on either side of the reaching movement.

Why might these initial, reactive, responses be decreased as learning proceeds? In the case of cocontraction, reduction likely occurs to decrease the metabolic cost of the movement (Huang and Ahmed 2014; Huang et al. 2012). However, the metabolic cost of increased visuomotor feedback gains would be very small, not even requiring attentional demands for hand motion (Reichenbach et al. 2014). On the other hand, the visuomotor feedback systems can be affected by distractors: producing incorrect responses to visual movement of objects. Therefore, high feedback gains may be limited to avoid increased responses to distractors.

In the second experiment, the visuomotor feedback gains were significantly larger in the unstable environment compared with the stable condition. Such feedback gain increases for unstable dynamics have been shown previously for stretch reflex responses during reaching (Franklin et al. 2007) and isometric tasks (Krutky et al. 2010). In unstable environments, uncertainty in the internal model of the dynamics and increased unpredictability overall is likely maintained, even after training. Therefore, in these environments, one expects overall increased cocontraction and stiffness (Burdet et al. 2001; Franklin et al. 2003), along with these higher feedback gains. Such changes allow rapid responses to any unpredictable movements due to the instability. Here, we show that we also find higher visuomotor feedback gains along with the previously shown stretch-dependent gains.

We examined the feedback gains over two intervals: the first corresponding to a rapid involuntary response (180–230 ms) and the second to a slower response (230–300 ms), which may either be involuntary or a mixture of involuntary and voluntary responses. The early interval was conservatively determined (Franklin and Wolpert 2008) using a voluntary reaction task (Day and Lyon 2000) to determine an interval that avoided any voluntary responses. There is some discussion (Franklin 2016) about whether these responses occur through a direct subcortical pathway through the superior colliculus (Reynolds and Day 2012) or a cortical pathway (such as through PMd). Different neural pathways likely produce responses at different delays relative to the initial perturbation and may also show different responses or controllability. We found that the visuomotor feedback gains were tuned appropriately to perturbations to either side of the reaching movement but that this differentiation occurred later than the initial response. It might be that this more complex “smart” feedback response indicates a more cortical pathway acting at a delay relative to the earliest response, while the initial response arises through a direct subcortical structure. However, any complex response depending on both internal models of the dynamics and body state might be expected to require intermediate neural layers and more synaptic connections increasing the response time. The separation of the analysis into two intervals does not suggest that each corresponds to a separate pathway involving different neural structures. It is simply performed to have one early interval in which we can avoid any voluntary response and a second interval in which we expect to see more gain modulation, which might also have a voluntary component.

Modulation of the feedback responses, according to the environmental dynamics, is appropriate when we consider the optimal feedback control theory of motor control (Scott 2004; Todorov 2004; Todorov and Jordan 2002). According to this theory, movement arises through the appropriate selection of time varying feedback gains for each task that minimizes a mixed cost function of terms such as accuracy and energy. This theory, therefore, emphasizes the critical role that feedback responses have in generating movement, and several studies have found evidence of such flexible, goal-directed feedback responses (Dimitriou et al. 2013; Liu and Todorov 2007; Nashed et al. 2014). Together, these predict that the feedback responses should adapt as we learn new skills (Scott 2012). Here, we have provided further support that the feedback responses are tuned according to the environment during adaptation. After adaptation to novel force fields, subjects normally only compensate for around 80% of the environmental dynamics (Howard et al. 2012; Sing and Smith 2010; Smith et al. 2006), such that the movement trajectories remain slightly curved. This has been suggested to arise as these curved trajectories assist in reducing the metabolic cost of the movements (Izawa et al. 2008), a consistent finding in the process of adaptation (Huang and Ahmed 2014; Huang et al. 2012). That is, Izawa and colleagues suggested that these S-shaped trajectories are the optimal solution to the force field, producing initial overcompensation to the force field when the forces are low and allowing the field to bring the hand back toward the target. In this optimal feedback control framework, the feedback gains must then be tuned according to the dynamics to produce the appropriate modifications in the trajectories. However, it has also been suggested that this lack of complete compensation to the environmental dynamics may arise due to the balance between the learning and forgetting rates of adaptation (Franklin et al. 2008; Scheidt et al. 2000; Smith et al. 2006), where this adaptation occurs through feedforward pathways. In this case, when compensation to the environmental dynamics is not complete, then the feedback responses may need to be adjusted to ensure that the movement can still reach the goal or successfully complete the task. These feedback gains, therefore, would be expected to be tuned to the dynamics, such that errors in the movement can be corrected appropriately. As changes in the feedback gains should be less costly metabolically compared with changes in muscle cocontraction, modulating the feedback pathways will provide an efficient mechanism for the control and adaptation of movement. Thus, both interpretations for partial force field adaptation suggest a role for feedback gains in adaptation to novel dynamics.

Our results reveal that feedback gain learning is, indeed, a critical part of dynamical adaptation, where feedback gains are tuned to the environmental dynamics. This opens up new questions in the mechanism of adaptation, specifically the mechanism by which the sensorimotor control system learns and tunes the feedback responses to the external environment.

## GRANTS

We thank the Wellcome Trust (WT091547MA and WT097803MA) and the Royal Society (Noreen Murray Professorship in Neurobiology to D. Wolpert) for support.

## DISCLOSURES

No conflicts of interest, financial or otherwise, are declared by the authors.

## AUTHOR CONTRIBUTIONS

S.F. and D.W.F. conceived and designed research; S.F. and D.W.F. performed experiments; S.F., D.M.W., and D.W.F. interpreted results of experiments; S.F., D.M.W., and D.W.F. drafted manuscript; S.F., D.M.W., and D.W.F. edited and revised manuscript; S.F., D.M.W., and D.W.F. approved final version of manuscript; D.W.F. analyzed data; D.W.F. prepared figures.
